# Loss of Stomatal Regulation Sensitivity to CO_2_ and Reduced Xylem Hydraulic Conductivity Contribute to Long‐Term Tree Decline and Mortality

**DOI:** 10.1111/gcb.70221

**Published:** 2025-05-20

**Authors:** Dario Martin‐Benito, Macarena Férriz, María Conde, Georg von Arx, Patrick Fonti, José Miguel Olano, Guillermo Gea‐Izquierdo

**Affiliations:** ^1^ Institute of Forest Sciences ICIFOR Inia‐CSIC Madrid Spain; ^2^ Department of Geography Indiana University Bloomington Indiana USA; ^3^ Swiss Federal Institute for Forest, Snow and Landscape Research WSL Birmensdorf Switzerland; ^4^ Oeschger Centre for Climate Change Research University of Bern Bern Switzerland; ^5^ iuFOR, EiFAB Universidad de Valladolid Soria Spain

**Keywords:** abiotic stress, canopy defoliation, drought‐induced decline, forest mortality, functional plasticity, global change, mixed forest

## Abstract

Increasing aridity is a major threat to forests worldwide. Understanding tree functional constraints under drought and their impacts on resilience and mortality among species is crucial to assess the impacts of global change on forests. We analyzed the long‐term drought and atmospheric CO_2_ responses in three Mediterranean co‐occurring species with differing drought tolerances ( *Pinus pinaster* < *Pinus pinea* < *Juniperus oxycedrus* ). In this mixed forest, *P. pinaster* exhibited widespread mortality and mistletoe infection, *P. pinea* showed scattered mortality, and *J. oxycedrus* showed no decline. Using tree‐ring data (1978–2016), we compared intrinsic water‐use efficiency (iWUE) and xylem hydraulic traits in healthy and non‐healthy individuals of both pine species and healthy junipers. Healthy *P. pinaster* trees produced a more hydraulically efficient xylem, with wider lumen tracheids, than non‐healthy trees, whereas *P. pinea* showed no anatomical differences between health statuses. Healthy *P. pinaster* displayed greater anatomical plasticity, adjusting hydraulic conductivity and cell‐wall thickness to water availability. Despite small differences in average iWUE, the response of iWUE to rising CO_2_ and drought differed between species and health statuses. *J. oxycedrus* and *P. pinea* showed steady iWUE increases, but *P. pinea* experienced periods of stagnation following an extreme drought, later recovering regardless of health status. In contrast, iWUE in *P. pinaster* plateaued for over 20 years after a decline‐inducing drought, particularly in non‐healthy, mistletoe‐infected trees. Differences in iWUE response to CO_2_ and anatomical plasticity to drought may explain the contrasting mortality patterns among these coniferous species. Our results suggest a long‐term decline spiral in *P. pinaster* induced by low hydraulic efficiency in drought‐induced defoliated trees and limited physiological responses to rising CO_2_ and drought. Increasing drought stress makes pine recovery increasingly unlikely.

## Graphical abstract

Increasing drought threatens forests, but tree species respond differently. We examined how long‐term drought and rising CO_2_ affect three Mediterranean tree species with different drought tolerances. *P. pinaster* showed widespread mortality, linked to reduced xylem hydraulic efficiency and a limited ability to adjust its water‐use efficiency under stress and a limited capacity to benefit from rising CO_2_. In contrast, *P. pinea* and *J. oxycedrus* showed higher plasticity in water‐use efficiency, which increased with increasing atmospheric CO_2_. Species differences in hydraulic traits and physiological plasticity helped explain the observed patterns of decline and mortality. Our results highlight how species‐specific responses to drought and atmospheric CO_2_ influence tree vulnerability under climate change.

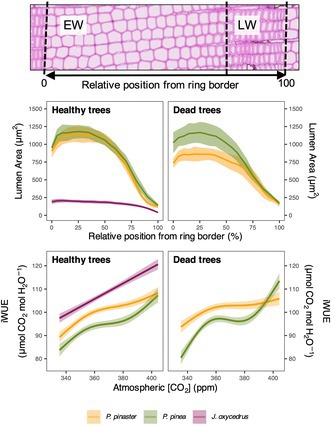

## 1 Introduction

Global warming has increased the frequency, intensity, and duration of drought episodes (Dai 2013). Extensive events of drought‐induced tree die‐off have occurred across many different forests worldwide (Allen et al. 2015; Hammond et al. 2022) and are expected to further increase under global change. Drought‐induced forest mortality is a complex process involving abiotic and biotic interactions, with drought acting as a long‐term predisposing factor (Manion 1981; McDowell et al. 2022). Interspecific and intraspecific differences in drought tolerance (e.g., resistance to cavitation, stomatal sensitivity, level of isohydricity) and susceptibility to secondary stressors, such as mistletoe or bark beetles, add further complexity to drought‐induced mortality (Klein 2015; McDowell et al. 2008; O'Brien et al. 2017). These differences may create a large mosaic of individual responses to drought that conditions the overall species and ecosystem response.

In response to drought, trees coordinate a myriad of functional traits in different organs (Mencuccini et al. 2015). Plants may close stomata to reduce transpiration and avoid hydraulic failure from embolism. Stomatal closure, however, reduces carbon uptake, negatively affecting growth (Choat et al. 2012; Martin‐StPaul et al. 2017) and other plant functions that may result in negative alterations of tree xylem (Adams et al. 2017; Hartmann et al. 2013). In contrast, maintaining transpiration rates to support water absorption may lead to tree water potentials below lethal thresholds and induce xylem hydraulic failure (Hacke and Sperry 2001), and ultimately result in tree die‐off (McDowell et al. 2008). Rising atmospheric CO_2_ concentrations may allow trees to maintain or increase photosynthetic rates (A) with reduced stomatal conductance (*g*_*s*_) at the leaf level and thus increase water use efficiency (Farquhar et al. 1989; Francey and Farquhar 1982; McCarroll and Loader 2004) and partly reduce the negative effects of drought (Keenan et al. 2013; Mathias and Thomas 2021; Saurer et al. 2004).

Xylem structures generally face a trade‐off between facilitating efficient water transport and reducing the risk of cavitation (Gleason et al. 2016; Tyree and Zimmermann 1983). In conifers, tracheid anatomical features crucial for hydraulic function, such as lumen area (LA) and cell wall thickness (CWT), are influenced by genetic, ontogenic, and climatic factors (Brodribb and Hill 1999; Bryukhanova and Fonti 2013; Cabon et al. 2020; Castagneri et al. 2018; Eilmann et al. 2009; Steppe et al. 2006). Trees often produce tracheids with larger lumens during periods of high water availability (Castagneri et al. 2018; Martin‐Benito et al. 2017) because larger tracheid lumens increase hydraulic conductance (Tyree and Zimmermann 1983). Thicker walls and higher resistance‐to‐implosion factors in tracheids (Hacke and Sperry 2001) correlate with safety against cavitation in some conifer families, including *Pinaceae* and *Cupressaceae* (Bouche et al. 2014; Gleason et al. 2016; Pittermann and Sperry 2006) but other factors such as length and density or pit architecture may affect their hydraulic safety more than LA or CWT (Bouche et al. 2014; Gleason et al. 2016; Pittermann et al. 2006; Sviderskaya et al. 2021). Cell walls represent a major carbon demand (Cuny et al. 2014, 2015), which is affected by water availability and temperature at different time scales (Fonti et al. 2013; Kirdyanov et al. 2003; Martin‐Benito et al. 2017; Pellizzari et al. 2016). Reduced carbohydrate availability may result in decreased carbon allocation to xylem reinforcement.

Pine‐juniper woodlands found across different regions globally have experienced severe drought‐induced mortality events, particularly affecting pine species, which are generally more isohydric and less drought‐tolerant than sympatric junipers (Allen and Breshears 1998; Breshears et al. 2009; Plaut et al. 2012). An illustrative case is the Mediterranean mixed conifer forests of Central Spain, composed of *Pinus pinaster* Ait., *Pinus pinea* L., and *Juniperus oxycedrus* L. These three species represent a gradient in isohydry, ranging from the more anisohydric *J. oxycedrus* to the more isohydric pines, although *P. pinea* shows less isohydric behavior than *P. pinaster* (Aranda et al. 2024). *Pinus pinaster* shows high levels of defoliation and experiences long‐term multidecadal decline and accelerated mortality (Férriz et al. 2021; Gea‐Izquierdo et al. 2019) aggravated by mistletoe infection, which increases whole‐tree transpiration (Bose et al. 2024; Yan et al. 2016; Zweifel et al. 2012). Populations of *P. pinaster* from xeric‐continental areas, like those in Central Spain, show lower plasticity and increased risk of hydraulic failure than more mesic populations (Ramírez‐Valiente et al. 2025). In contrast, *P. pinea* experiences a short‐term multiannual decline before mortality, whereas *J. oxycedrus* shows no symptoms of decline. Both *P. pinea* and *J. oxycedrus* show much more abundant regeneration than *P. pinaster* . Because of the different drought‐tolerance strategies exhibited by these three species (Aranda et al. 2024; Férriz et al. 2023) and because the different dynamics experienced by them are likely produced by increasing stress under climate change (Férriz et al. 2021; Gea‐Izquierdo et al. 2019), these patterns of mortality and regeneration are likely to be exacerbated with future aridity intensification. These conditions may be particularly detrimental to *P. pinaster* because of its low stomatal sensitivity to CO_2_ (Picon et al. 1996; Sánchez‐Gómez et al. 2017) which may limit its performance under drought (Aranda et al. 2024).

Comparative multiproxy studies of wood anatomy and tree‐ring δ^13^C‐derived iWUE between healthy and non‐healthy trees provide insights on the declining process and on community dynamics under global change (Hereş et al. 2014; Levanič et al. 2011; Pellizzari et al. 2016; Puchi et al. 2021). In some species, healthy trees may optimize water transport by producing wider lumens with lower carbon investment towards xylem reinforcement than declining trees (Hereş et al. 2014; Petrucco et al. 2017), while healthy trees of other species may reinforce tracheids to reduce vulnerability to cell implosion compared to non‐healthy trees (Levanič et al. 2011; Petrucco et al. 2017; Voltas et al. 2013). Water use efficiency may differ between healthy and non‐healthy trees depending on species, site, or levels of stress reached (Gessler et al. 2018). Tighter stomatal control in declining trees than in healthy trees may increase their iWUE (Voltas et al. 2013), but also increased water transpiration of remaining leaves in declining trees may decrease their iWUE compared to healthy individuals (Colangelo et al. 2017). The combined analysis of complementary functional traits and their plasticity to variable conditions (Nicotra et al. 2010) contributes to assessing intra‐ and inter‐specific different strategies to cope with water stress and identify factors that induce forest decline and tree mortality (Gessler et al. 2018; Mencuccini et al. 2015; Pellizzari et al. 2016; Puchi et al. 2021). Phenotypic plasticity may be expressed as high responses or sensitivity of functional traits to drought or atmospheric CO_2_ allowing trees to regulate transpiration while maintaining a positive carbon balance and ultimately determining individual and species survival. Species responses and adaptation to climate change are critical for the survival and distribution of forest communities. Greater plasticity in drought tolerance traits, including iWUE and xylem anatomy, adjusting these traits for higher drought resistance may increase overall performance (Challis et al. 2022; Corcuera et al. 2011; Rosas et al. 2019) and contribute to decreasing or delaying tree damage and mortality under severe drought.

In this study, we investigated the coordinated responses of physiological and wood hydraulic traits to drought and CO_2_ and their implications for the ongoing forest decline and mortality among three co‐occurring Mediterranean conifers (Férriz et al. 2021). We analyzed radial growth, iWUE, and xylem anatomical trait patterns to provide a comprehensive interpretation of the anatomical and physiological mechanisms driving forest decline and mortality, with a long‐term perspective using tree‐ring proxies, and to explore whether plasticity in these functional traits reflects the different mortality patterns observed in our study forest. Our main hypothesis was that low plasticity in species' functional traits, expressed as sensitivity to environmental variables, predisposes trees to reduced overall performance, leading to decline and accelerated mortality (Aranda et al. 2024; Corcuera et al. 2011; Rosas et al. 2019; Sánchez‐Salguero et al. 2018). Specifically, we hypothesized that: (1) more drought‐tolerant species develop reinforced, but hydraulically less efficient xylems; (2) healthy pines maintain higher long‐term hydraulic potential conductivities and tighter stomatal control (increased iWUE) than non‐healthy trees; (3) lower sensitivity to drought in xylem anatomical traits and iWUE in unhealthy trees reflects their reduced plasticity to adapt to climatic variability and endure increased stress under climate change; (4) increasing atmospheric CO_2_ has alleviated drought stress more on species and individuals not experiencing decline.

## 2 Materials and Methods

### 2.1 Study Site and Tree Sampling

**FIGURE 1:**
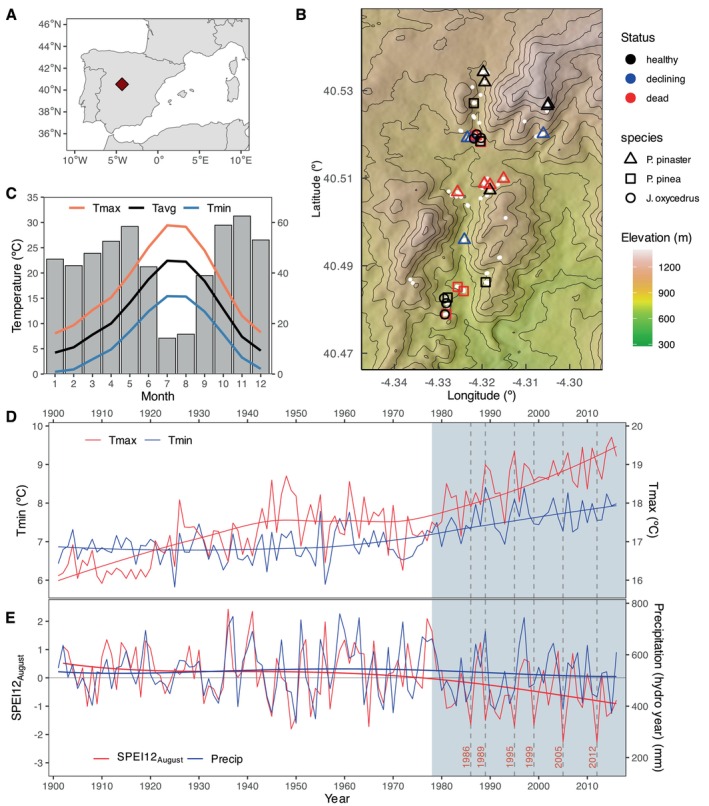
Research location and climate of study area. (A) Location of the study area in Central Spain. (B) Detail of study site with distribution of all sampled trees (white dots) and those selected for anatomical and isotopic analysis (colour symbols). (C) Climate diagram showing mean temperature and precipitation for the study location from closest grid point CRU TS 4.01 data set. (D) Maximum and minimum temperature between 1900 and 2016. (E) Precipitation for the hydrological year (previous September through current August) and SPEI12 in August between 1900 and 2016. In (C) and (D), vertical lines show the six driest years in the period 1978–2016, all with August SPEI12 < −1.51. In (D) and (E), temperature and hydroclimate trends are highlighted. Map lines delineate study areas and do not necessarily depict accepted national boundaries.

Our study site was a mixed forest in the Iberian Central Mountain Range (40.5º N; 4.29º W) (Figure 1). The climate is Mediterranean, i.e., with annual summer drought and a wet, cool season, with a mean annual temperature of 12.3°C and a mean annual precipitation sum of 533 mm for the period 1901–2016. The forest is dominated by *Pinus pinaster* and *P. pinea* with abundant *Juniperus oxycedrus* and *Quercus ilex* L. The area represents the local lower altitudinal limit of *P. pinaster* and the upper limit of *P. pinea* . In forests where these two pine species coexist within the studied altitudinal range, *P. pinaster* shows decline symptoms at individual (high defoliation, mistletoe *Viscum album* L. infestation) and population levels (high annual mortality rates, lack of regeneration) whereas *P. pinea* shows only scattered dead individuals and more abundant regeneration (Férriz et al. 2021; Gea‐Izquierdo et al. 2019). In the area, *J. oxycedrus* generally exhibits good health, abundant regeneration, and very few dead individuals large enough to be sampled. These three species represent a drought tolerance gradient *P. pinaster* < *P. pinea* < *J. oxycedrus* derived both from their biogeographical distribution and species physiological performance under drought (Aranda et al. 2024; Férriz et al. 2021; Martinez‐Vilalta et al. 2004).

In 2016 and 2017, we selected and sampled 20–22 trees per health status and species for a total of 60 *P. pinaster* (20 healthy, 20 declining and 20 dead trees), 42 *P. pinea* (22 healthy and 20 dead trees) and 22 healthy *J. oxycedrus* across an elevational gradient between 790 and 1200 m a.s.l where the three species coexist (Figure 1). For further information, see Férriz et al. (2021) and Gea‐Izquierdo et al. (2019). Throughout this work, we refer to dead trees as those that were dead at the time of sampling. Trees were sampled within a few months of their death and while they still had dry needles. For each tree, we measured diameter at breast height (DBH) and tree height. We assessed health status based on crown defoliation and levels of mistletoe infestation (Dobbertin 2005; Férriz et al. 2021; Gea‐Izquierdo et al. 2019). Crown defoliation was assessed on a scale from 0 to 4, where 0 indicated a full crown and 4 represented complete defoliation (dead tree). Mistletoe infection was similarly graded from 0 (absence of mistletoe) to 4 (heavy infection affecting multiple branches; Galiano et al. 2010). Two mistletoe species are present in conifers in the study area: *Viscum album* on *P. pinaster* and *Arceuthobium oxycedri* (DC.) M.Bieb. on *J. oxycedrus* . Mistletoes do not seem to infest *P. pinea* (López Sáez and Sanz de Bremond 1992) and were not observed on that species in our study.

From each tree, we collected three cores with an increment borer. All three cores per tree were processed until annual rings were clearly visible. One core was glued onto wooden boards and sanded. The other two were not glued, and their surface was prepared with a sledge microtome to avoid cross‐contamination of wood dust between different annual rings (Gärtner and Nievergelt 2010). On these three cores, annual ring widths were dated and measured using a LINTAB measuring table connected to the TSAP software (Rinn 2003). Visual crossdating was statistically verified with COFECHA (Holmes 1983). From ring width and DBH corrected for bark thickness, we estimated tree annual growth as basal area increments (BAI, cm^2^ year^−1^) as BAI = π·(*R*_*t*_^2^− *R*_*t*−1_^2^), where *R*_*t*_ and *R*_*t*−1_ are the radius of a tree at year *t* and *t*−1, respectively. For a detailed analysis of the temporal trends in BAI, see Férriz et al. (2021). For analyses of tree carbon isotopic ratios and wood anatomical traits, we selected a subset of five representative trees per species and health status (30 trees in total).

### 2.2 Quantitative Wood Anatomy

**FIGURE 2:**
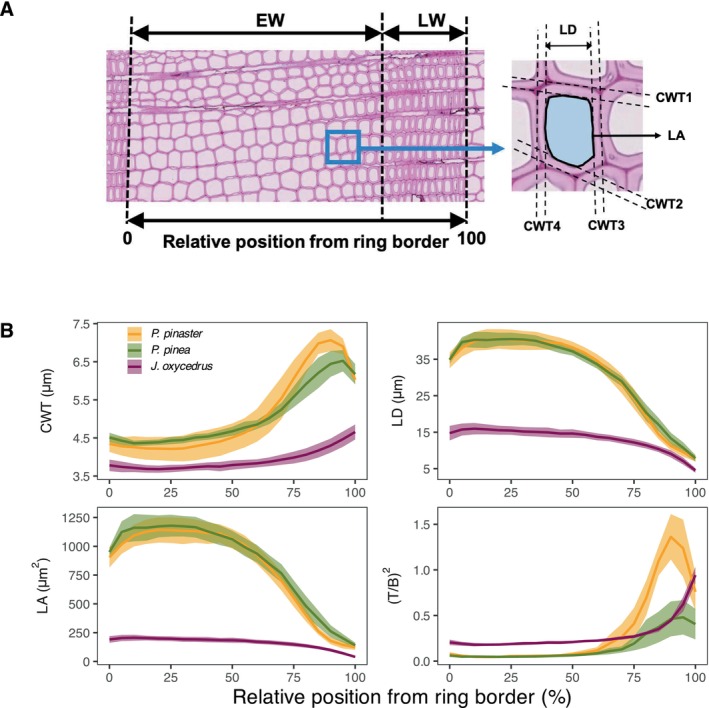
Xylem anatomical parameters along an annual growth ring. (A) Image of a tree‐ring micro section in *Pinus pinaster* with the two main ring sections earlywood (EW) and latewood (LW) and cell anatomical parameters measured including lumen diameter (LD), lumen area (LA) and cell‐wall thickness (CWT) in radial and tangential directions. (B) Mean anatomical parameters along the growth ring for all healthy individuals of the three species (5 trees per species): CWT estimated as the mean of four CWT per cell; lumen diameter (LD), lumen area (LA); resistance to implosion factor [(T/B)^2^]. Shaded areas around the mean represent ±1.96 SEM (standard error of the mean).

From the subset of 5 cores from each species and health status (30 trees in total), we developed and analyzed time series of wood anatomical traits for the period 1978–2016 (39 years). Cores were divided in sections 1.5–2.0 cm in length and embedded in paraffin. From each block, we cut transversal microsections 12‐μm thick using a rotary microtome (RM2245, Leica, Heidelberg, Germany). Microsections were stained with safranin and Astra blue, permanently fixed, and imaged with a slide scanner (Axio Scan.Z1, Zeiss, Jena, Germany) at a resolution of 2.27 pixels per μm. Details on sample preparation are described in Fonti et al. (2025). In those images, we measured the total number of tracheids per ring, cell‐wall thickness (CWT) in radial and tangential directions, lumen radial diameter (LD) lumen area (LA) and theoretical xylem hydraulic conductivity (KH) using the ROXAS software (Prendin et al. 2017; Von Arx and Carrer 2014). For all analyses, we selected the 15–20 complete tracheid rows, including the highest number of tracheids within each annual ring using the *RAPTOR* package in R (Peters et al. 2018). The implosion‐safety factor was estimated as (T/B)^2^, with T being cell‐wall thickness and B the conduit diameter (Hacke et al. 2001) (Figure 2). Tracheids were classified into either earlywood (EW) or late wood (LW) based on Mork's index (Denne 1989): EW cells with LD ≥ 4·CWT/LD, whereas LW cells with LD ≤ 4·CWT/LD.

### 2.3 Carbon Isotope Analyses

We analyzed the carbon isotope ratio (δ^13^C) annually from 5 trees in each health status and species (30 trees total) for the same period used for anatomical analysis (1978 and 2016). Cores were selected according to the: (i) absence of visible damages, such as missing parts, (ii) high correlation (≥ 0.70) with its species and health status master chronologies, (iii) rings wide enough to have enough wood mass to conduct isotopic analysis (> 1 mg). In both pine species, we analyzed all individual rings in the period 1978–2016. In *J. oxycedrus* , due to some very narrow rings, we selected one tree with wide rings to determine δ^13^C annually (1978–2016) and for the other four junipers, we pooled rings every 5 years to obtain a carbon isotopic signal representative of the species. Individual rings were separated with a scalpel under a microscope to reach the minimum wood mass required for δ^13^C analysis. Rings were individually powdered in a mixer mill MM 300 (Retsch, Haan, Germany). From each sample, we encapsulated 1 mg in tin foil capsules and analyzed stable C isotopic ratios by continuous flow Isotope Ratio Mass Spectrometer (IRMS) at the Stable Isotope Facility, UC Davis (CA, USA). Values of δ^13^C_tree_ were estimated relative to the standard Vienna Pee Dee Belemnite (VPDB):

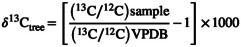




From δ^13^C_tree_, we estimated carbon isotope discrimination rates (Δ^13^C), as:

∆C13=δ13Cair−δ13Ctree1+δ13Ctree/1000

where δ^13^C_air_ is the C isotopic ratio in atmospheric air. We combined annual δ^13^C_air_ values for the period 1978–2003 from (McCarroll and Loader 2004) and from Mauna Loa (https://gml.noaa.gov/dv/iadv/) for the period 2004–2016. Thus,

$$c_{i}=c_{a}\frac{\delta^{13}C_{\text{tree}}-\delta^{13}C_{\mathrm{air}}+a}{b-a}$$

Intrinsic water‐use efficiency (iWUE), defined as the ratio between net photosynthetic rates (*A*_*n*_) and stomatal conductance to water vapour (*g*_*s*_), can be estimated as:

(1) $$\text{iWUE}=\frac{A_{n}}{g_{s}}=\frac{\left(c_{a}-c_{i}\right)}{1.6}$$

where 1.6 is the ratio of *g*_*s*_ to stomatal conductance for CO_2_ (Farquhar et al. 1989), *c*_*a*_ and *c*_*i*_ are the partial pressures of CO_2_ in the atmospheric air and in the intercellular space of the leaf, respectively. From tree‐ring Δ^13^C, iWUE can be approximated as:

(2) iWUEi=ca·b−∆C131.6·b−a

where *a* is the discrimination against ^13^CO_2_ during stomatal diffusion (≈ −4‰) and *b* is the net fractionation of CO_2_ due to discrimination by Rubisco during carboxylation (≈ −27‰) (Francey and Farquhar 1982).

### 2.4 Non‐Structural Carbohydrate Analysis

To analyze the content of non‐structural carbohydrates (NSC), we extracted 5‐cm‐long wood cores from each tree in winter 2017–2018. Cores were microwaved for 3 min to stop enzymatic activity, later kept at −18°C and freeze‐dried. After removing bark and phloem, subsamples of ~2 cm of sapwood were milled. We analyzed NSC following the anthrone method (Olano et al. 2006; Quentin et al. 2015) separating them into soluble sugars and an insoluble fraction (starch) expressed as a percentage of dry matter.

### 2.5 Climate Data and SPEI

To explore the effects of climate on growth, xylem isotopic proxies and anatomical variables, we used monthly precipitation and temperature (1900–2016) obtained from the closest grid point in the CRU TS 4.01 dataset (Harris et al. 2014). We estimated the multiscalar drought index Standardised Precipitation‐Evapotranspiration Index (SPEI) (Vicente‐Serrano et al. 2010) for periods of 3, 6, and 12 months with the *SPEI* R package (Beguería and Vicente‐Serrano 2017). The six driest years with the lowest SPEI12 (i.e., annual drought) in August (1986, 1989, 1995, 1999, 2005, and 2012; all with SPEI12 ≤ −1.51) were among the eight driest years since 1900 (Figure 1). We selected SPEI12 in August because it reflects the climatic conditions experienced by the trees when most growth and carbon allocation occur and to which trees show the highest climate‐growth correlations (see results).

### 2.6 Data Analysis

We analyzed mean differences in anatomical traits between species and health status using linear mixed models and the R package *lme4* (Bates et al. 2015) to account for the lack of independence caused by repeated measurements done on the same trees. In these mixed models, we included individual tree height as a covariate to account for the potential influence of tree height on xylem anatomical variables, particularly conduit lumen diameter and area (Anfodillo et al. 2006; Losso et al. 2018).

We calculated correlations between radial tree growth, anatomical traits, Δ^13^C and iWUE within species and status and monthly climate variables between October of the year prior to growth to September of the current year for the period 1978–2016 using the treeclim R package (Zang and Biondi 2015). To refine these analyses, we compared the distributions of LA and CWT along the annual growth ring for dry, average, and wet years within each species and health status combination with two‐sample Kolmogorov–Smirnoff tests. Because of the high uncertainty in crossdating juniper tree rings and the pooling performed for Δ^13^C analysis in this species, we did not run an analysis of the interannual variability of juniper traits in relation to climate. We also explored the resistance, resilience, and recovery of BAI and iWUE, as well as the recovery period to the most extreme droughts identified during the study period (Lloret et al. 2011; Schwarz et al. 2020).

To investigate the effects of atmospheric CO_2_ concentration and drought (estimated as SPEI12 in August) on intrinsic water‐use efficiency (iWUE), we used generalized additive models (GAMs) to allow for non‐linear relationships between iWUE and independent variables (Hastie and Tibshirani 1986). We tested whether the CO_2_‐iWUE relationship differed among health status and CO_2_ concentrations across the tree species ( *P. pinaster* , *P. pinea* , and *J. oxycedrus* ) with the following model per species:

$$\begin{array}{cc} \text{iWUE}\; & =\beta_{0}+\beta_{1}\times \text{Status}+s\left({\mathrm{CO}}_{2}\right)+s\left({\mathrm{CO}}_{2},\mathrm{by}\;\text{Status}\right)\\ & +s\left(\text{SPEI}12\right)+s\left(\text{SPEI}12,\mathrm{by}\;\text{Period}\times \text{Status}\right)+\epsilon \end{array}$$

where β_0_ is the intercept for the reference health status group; β_1_ is the linear effect for the other health status groups; *s*(CO_2_) and *s*(SPEI12) were the global smooth terms for CO_2_ and drought, respectively; *s*(CO_2_, by Status) the smooth term for the interactions between CO_2_ and health status; and *s*(SPEI12, by Period × Status) represents the smooth term for the effect of drought varying by the interaction between health status and period (i.e., before or after growth decline in pines) and *ϵ* is the error term. These two different periods in *P. pinaster* (before or after 1995) and in *P. pinea* (before or after 2005) were included in the models for SPEI to test for the potential effect of unknown variables other than CO_2_ and drought. Periods before and after growth decline were tested for SPEI because it is not a continuously increasing variable like CO_2_. The smooth effects of CO_2_ and SPEI12 were fitted with four knots to allow flexible responses to differ between health status and/or periods while keeping enough rigidity to avoid overfitting while maintaining ecologically realistic responses such as the expected constantly increasing iWUE with rising CO_2_ from Equation (2) and observations (Mathias and Thomas 2021; Silva and Horwath 2013). We used the Akaike Information Criteria (AIC) and deviance explained to assess the performance of different models fitted with maximum likelihood (ML). Once a model was selected, it was refitted with restricted maximum likelihood (REML). GAMs were fitted using the *mgcv* package in R (Pedersen et al. 2019; Wood 2017). We analyzed potential temporal changes in the iWUE response to CO_2_ across health status for each species by estimating the slopes of the iWUE‐CO_2_ relationship (ΔiWUE/ΔCO_2_) as the first derivatives of the smooth CO_2_ terms (Simpson 2018) using the *marginaleffects* package in R (Arel‐Bundock et al. 2024).

## 3 Results

### 3.1 Growth and iWUE Temporal Trends

**FIGURE 3:**
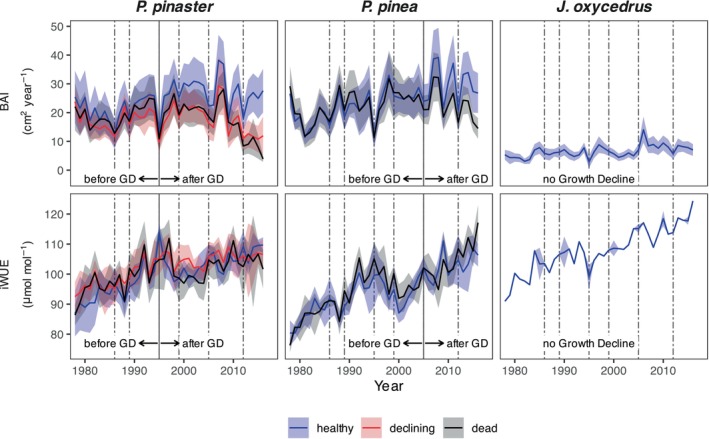
Annual growth in basal area increment (BAI) and intrinsic water‐use efficiency (iWUE) for the period 1978–2016. Lines represent average values and shaded areas confidence intervals around the mean (±1.96⋅SEM, standard error of the mean). Vertical lines show the 6 driest years (lowest August SPEI12, see Figure 1): 1986, 1989, 1995, 1999, 2005, 2012. Samples size was 5 trees per species and health status in all cases. The periods before and after growth decline (GD) are highlighted for *P. pinaster* and *P. pinea* (i.e., 1995 and 2005, respectively). *Juniperus oxycedrus* showed no growth decline during the study period.

**FIGURE 4:**
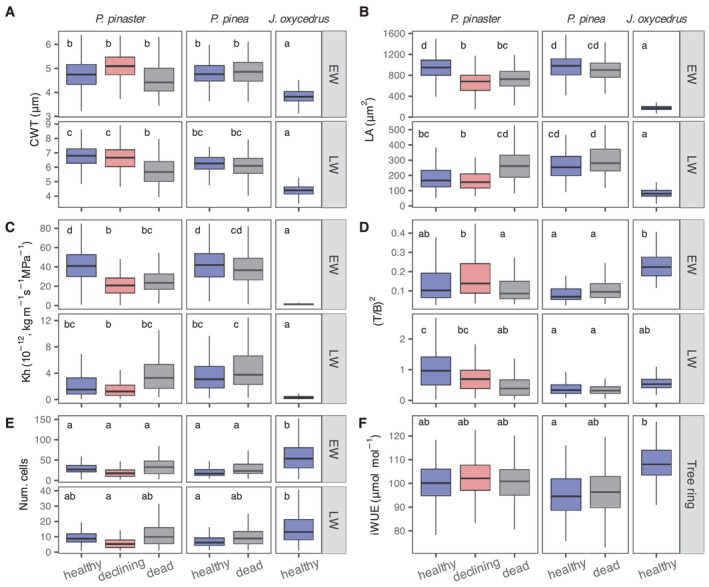
Xylem anatomical and isotopic variables measured for each species and health status. Values are means of 5 trees per species and health combination averaged over the study period (1978–2016). Variables are: (A) CWT, cell‐wall thickness; (B) LA, lumen area; (C) Kh, theoretical xylem hydraulic conductivity; (D) (T/B)^2^, resistance‐to‐implosion factor; (E) Num. cells, radial number of cells; (F) iWUE, intrinsic water‐use efficiency. Section, either earlywood (EW), latewood (LW) or the entire tree ring. Different letters indicate significant differences between means within the same variable and ring section using linear mixed effects models to account for the lack of independence of data within each individual and temporal autocorrelation. Tree height was also included in mixed models to account for the potential influence of tree height on xylem anatomical variables. Outliers were included in all analysis (linear mixed models and boxplot statistics) but are not displayed in the figure for clarity.

On average, *Pinus pinea* trees were older than *J. oxycedrus* and *P. pinaster* trees (Table 1). *P. pinaster* trees were the tallest, whereas *P. pinea* had the largest DBHs, with *J. oxycedrus* presenting the lowest heights and DBHs of all three species. Tree growth (BAI) in both pine species was similar and higher than in junipers (Figure 3). Tree growth of non‐healthy *P. pinaster* trees (declining and dead individuals) started diverging from that of healthy individuals after the extreme drought in 1995: while healthy trees maintained relatively stable average growth, non‐healthy trees progressively decreased (Figure 3). In contrast, in *P. pinea* , the growth of dead trees declined after the drought year 2005 and diverged from healthy trees. Juniper BAI constantly increased throughout the study period (see Férriz et al. (2021) for further details). In both pine species, iWUE increased from 1978 to 1995, then decreased until around 2000 (Figure 3). After the early 2000s, iWUE remained stable in *P. pinaster* but continued to rise in *P. pinea* . Overall, *J. oxycedrus* showed the fastest and most steady iWUE increase of the three species for the study period. Despite the different trends, mean iWUE values did not differ significantly between health classes (Figure 4; Table S1).

**TABLE 1:** Descriptive characteristics of sampled trees.

Species	Status	DBH (cm)	Height (m)	Age (year)	Mistletoe^a^	Defoliation
*P. pinaster*	Healthy	54.2 (11.8)	18.9 (4.5)	83.2 (25.6)	0.4 (0–2)	1 (1–1)
	Declining	45.6 (3.9)	16.8 (3.8)	107.8 (46.7)	1 (0–3)	2.4 (2–3)
	Dead	36.5 (6.1)	12.2 (1.2)	62.2 (4.9)	2.6 (0–4)	4 (4–4)
*P. pinea*	Healthy	65.5 (9.2)	15.2 (4.1)	155.6 (38.7)	0 (0–0)	0.4 (0–1)
	Dead	64.3 (11.7)	14.1 (2.8)	113.8 (17.5)	0 (0–0)	4 (4–4)
*J. oxycedrus*	Healthy	24.4 (6.7)	7.8 (0.3)	73.8 (25)	0 (0–0)	0 (0–0)

*Note:* Values are means and standard deviations (in parentheses), except for mistletoe and defoliation where range is given in parentheses. Mistletoe and defoliation were estimated in five categories (0–4, lowest to highest). Sample size was 5 trees in all cases.

Abbreviation: DBH, diameter at breast height.

^a^ Potential mistletoe species: *Viscum album* on *P. pinaster* and *Arceuthobium oxycedri* on *J. oxycedrus* .

### 3.2 Variability in Xylem Functional Traits in Healthy Trees of All Species

Tracheid lumen area (LA), lumen diameter (DH), and wall thickness (CWT) were smaller in *J. oxycedrus* than in pines (Figures 2 and 4). These differences were particularly apparent in the earlywood, where LA of juniper cells was 5 times smaller than those in healthy pines. In general, tracheid CWT and LA dimensions were similar in both pine species (Figures 2 and 4). Consequently, xylem potential conductivity (Kh) was 3 times higher in healthy trees of both pine species than in junipers. The resistance‐to‐implosion factor ([T/B]^2^) was similar in earlywood tracheids of both pine species, but smaller than in junipers, particularly when compared with *P. pinea* . The largest [T/B]^2^ was observed in latewood tracheids of healthy *P. pinaster* . In contrast, junipers produced 2–3 times more tracheid cells per annual ring than pines. Carbon isotopic discrimination (Δ^13^C) was highest in *P. pinea* trees followed by *P. pinaster* and then *J. oxycedrus* . Soluble sugars, the largest NSC pool, were significantly higher in both pine species than in *J. oxycedrus* , whereas starch content was similar in the three species (Figure S1).

### 3.3 Variability in Xylem Functional Traits Between Different Health Statuses in Pines

Healthy *P. pinaster* individuals had significantly larger lumens (both LA and DH) in the earlywood and thicker CWT in the latewood than dead and declining trees (Figure 4; Table S1), particularly in the most recent years within the analyzed period (Figure S2). These differences resulted in higher potential conductivity in health*y P. pinaster*, but also in higher implosion safety factors in the LW than dead and declining trees due to their thicker latewood CWT. In general, tracheid dimensions of declining *P. pinaster* trees were intermediate between those of healthy and dead individuals. In contrast, average xylem anatomical traits were similar between healthy and dead *P. pinea* . The soluble sugar fraction of NSC in healthy trees was significantly higher than in dead trees, but not the starch fraction, which was slightly higher in dead *P. pinea* trees (Figure S1).

All species and health status showed inverse relationships between lumen area (LA) and cell wall thickness (CWT) (Figure S3). Ring width (RW) was mostly influenced by the number of cells (Ncells). Cross‐correlations between functional traits were in general higher and more often significant in healthy than in non‐healthy individuals, particularly in *P. pinea* . Correlations between iWUE and Ncells were positive in healthy *P. pinea* but negative in all *P. pinaster* and dead *P. pinea* . In contrast, iWUE was not correlated with hydraulic traits (LA or KH) or the resistance to implosion factor (T/B)^2^.

### 3.4 Effects of Climate and Drought on Xylem Anatomical and Isotopic Functional Traits

Spring–summer drought was the most influential climate factor for tree growth, iWUE, and xylem anatomical variables (Figure S4). In both pine species, iWUE increased with temperatures and drought stress (negative correlation with SPEI) during spring and summer. In contrast, the response of anatomical traits to climate was more variable, healthy trees being more sensitive to climatic variability. Resistance and resilience of BAI to drought were similar in all species, with the lowest resistance observed to the extreme 1995 drought (Figure S5A). Trees of all health statuses in both pine species showed similar resilience indices for BAI, but resistance and resilience for non‐healthy *P. pinaster* decreased with time while the recovery period increased, particularly for the latest drought in 2012 (Figure S5A). In contrast, iWUE of healthy *P. pinaster* was more resistant than that of non‐healthy trees but showed lower recovery (Figure S5B). In general, non‐healthy pine trees had longer recovery periods for their pre‐drought iWUE levels than healthy trees.

**FIGURE 5:**
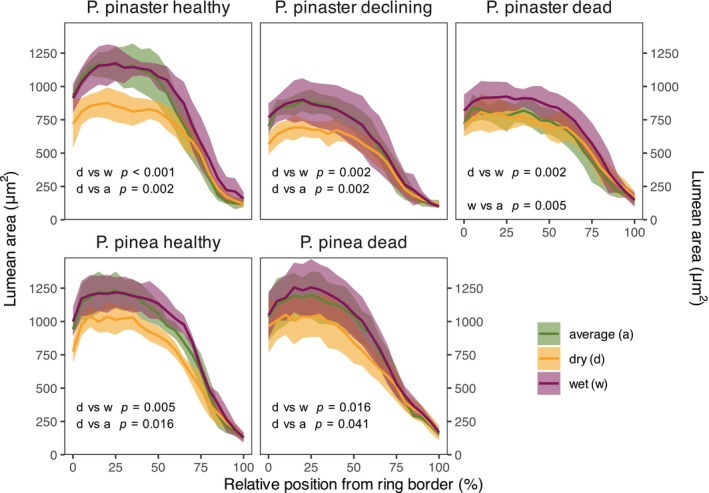
Variation of tracheid lumen area (LA) along the annual growth ring of both pine species for the 6 driest, 6 wettest and 6 average years i.e., those closest to zero (estimated from August SPEI12 between 1976 and 2016; see Figure S1). Thick lines represent average values and shaded areas represent confidence intervals around the mean (±1.96 SEM, standard error of the mean). Kolmogorov–Smirnoff tests were used to check for significant differences between results for dry (d), average (a) and wet (w) years within each species and health status combination. Results shown when significant for two‐sample Kolmogorov–Smirnov tests (α ≤ 0.05).

Drought stress reduced earlywood LA and latewood CWT (positive correlations with SPEI) in both pine species (Figure S4). The intra‐ring analyses between contrasting drought years also showed strong negative effects of drought on LA (Figure 5) and positive, but smaller effects on CWT (Figure S6), particularly on healthy trees. Tracheids formed during extreme dry years exhibited reduced LA in the first ~75% of the ring compared to wet or normal years, with larger reductions in healthy pines (Figure 3). During dry years, trees of all health statuses had similar LA sizes, but healthy trees had much larger LA during wet and normal years. In CWT, significant but small differences were observed only in healthy and declining *P. pinaster* with thicker CWT in the earlywood and thinner in the latewood during dry years compared to wet or normal years (Figure S6).

### 3.5 Response of iWUE to CO_2_ and Drought Among Species and Health Status

**FIGURE 6:**
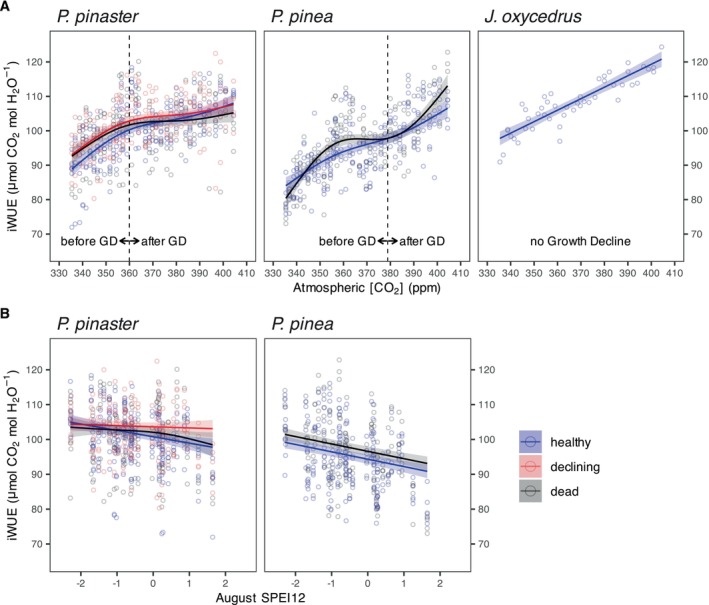
Partial effects of (A) atmospheric [CO_2_] and (B) drought (August SPEI12) on each species' intrinsic water‐use efficiency (iWUE) in 1978–2016. Circles show annual observations per tree and health status in each species. Solid lines represent predicted iWUE values from each GAM model when drought (SPEI12 in August) in (A) and atmospheric [CO_2_] in (B) are set to their mean value (i.e., the partial effects). Shaded areas represent confidence intervals (±1.96·standard error). Health status had a significant effect on both pine species. Vertical dash lines in (A) for *P. pinaster* and *P. pinea* represent atmospheric [CO_2_] of the year when growth decline started (1995 and 2005, respectively). *Juniperus oxycedrus* showed no growth decline during the study period. Summary for these models can be found in Table S3.

**FIGURE 7:**
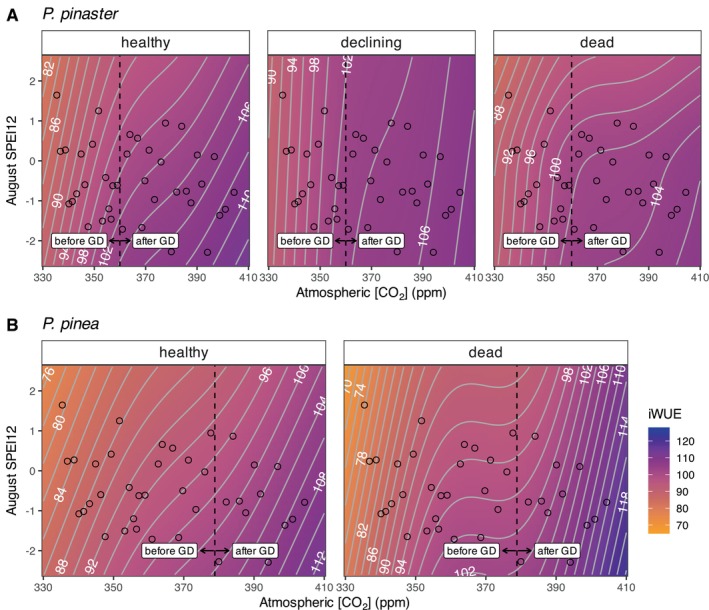
Interactions between health status and August SPEI12 and atmospheric [CO_2_] on iWUE for (A) *Pinus pinaster* and (B) *Pinus pinea* . The interactions shown represent predictions of iWUE from species‐specific models (Tables S1 and S2) across the range of observed August SPEI12 and atmospheric [CO_2_] for the study period (1978–2016). Vertical dash lines in (A) *P. pinaster* and (B) *P. pinea* represent atmospheric [CO_2_] of the year when growth decline (GD) started (1995 and 2005, respectively). Summary for these models can be found in Table S3.

The best GAMs for junipers and both pine species reflected a significant relationship of iWUE with CO_2_, which was different for the different health statuses of both pine species (Tables S2 and S3). As expected, iWUE increased with increasing atmospheric CO_2_ concentrations in all species and health statuses, but there were important differences in the magnitude and variability of these trends (Figure 6A; Tables S2 and S3). In junipers, iWUE increased steadily and linearly (29%) with increasing CO_2_ for the entire period, but its overall increase was lower (from iWUE ~90 to ~120 μmol mol^−1^) than in any of the pine species (from iWUE ~70 to ~120 μmol mol^−1^). In *P. pinaster* , for a ~7.5% increase in CO_2_ concentrations between 1978 and 1995 (335–360 ppm), iWUE increased by 32%, 14%, and 21% for healthy, declining, and dead trees, respectively. Later, between 1996 and 2016, despite a ~11.5% increase in CO_2_ (360–404 ppm), iWUE remained rather constant in non‐healthy trees (mean increases < 1%) with 5% increases in healthy trees (Figures 6A and 7A). Thus, the slopes of the iWUE‐CO_2_ relationship (ΔiWUE/ΔCO_2_) decreased between 335 and 360 ppm and were very close to zero in non‐healthy *P. pinaster* trees above 360 ppm [CO_2_] (Figure S7). In contrast, in *P. pinea* , iWUE increased with CO_2_ until 360 ppm in both healthy (25%) and dead trees (38%). Between 1995 and 2005, when growth in dead trees started declining, iWUE plateaued in dead trees and increased more slowly in healthy trees (Figure 6A and Figure S7). After 2005 (> 380 ppm [CO_2_]), iWUE increased again in *P. pinea* (10% and 15% in healthy and dead trees, respectively) until 2016.

Drought stress increased iWUE in both pine species, particularly in *P. pinea* (Figure 6A). However, the influence of drought on iWUE was only significantly different between health statuses in *P. pinaster* , with higher sensitivity in healthy trees (Figure 6A). Periods before and after growth decline in both pine species showed no significant differences in the response of iWUE to drought, likely because temporal differences were accounted for by CO_2_ (Figure 7; Table S2). Thus, trees of both pine species reached their highest iWUE under the combined effect of elevated CO_2_ and strong drought conditions (Figure 7). However, *P. pinea* was more responsive to these conditions than *P. pinaster* , and healthy *P. pinaster* trees more than dead and declining trees (Figure 7).

## 4 Discussion

Our retrospective analysis of intrinsic water‐use efficiency (iWUE) and variability of xylem traits in three co‐occurring Mediterranean conifer species with different drought tolerances ( *Pinus pinaster* < *Pinus pinea* < *Juniperus oxycedrus* ) showed the importance of the long‐term responses at the stomatal and xylem level towards tree survival and different mortality syndromes under drought stress. Plasticity in the response of functional traits, such as iWUE and xylem anatomical architecture, to climate and other environmental factors suggests that trees' survival is largely determined by their ability to adjust to changing conditions. These differences among species and health conditions set a foundation for understanding the diverse mortality processes observed in tree species.

### 4.1 Loss of Sensitivity in iWUE to CO_2_ Associated with *P. pinaster* Decline

Overall, iWUE (A/g_s_) increased with CO_2_ concentrations across all species and health statuses over our 39‐year study period (1978–2016) in line with the global increase in iWUE over the recent decades (Frank et al. 2015; Keenan et al. 2013; Mathias and Thomas 2021; Saurer et al. 2004, 2014). Junipers showed the highest mean iWUE of the three species and higher plasticity to CO_2_ (a steady increase in iWUE), which allowed them to benefit from rising CO_2_ despite increases in drought stress. In contrast, both pine species showed complex temporal patterns and environmental influences. Mean iWUE over the study period was similar across different health statuses in *P. pinaster* and *P. pinea* , in contrast to previous reports of healthy trees having higher (Hentschel et al. 2014) or lower iWUE (Petrucco et al. 2017; Voltas et al. 2013) than non‐healthy trees. However, the temporal trends of iWUE differed among pine species, with iWUE in *P. pinea* increasing faster with CO_2_ than *P. pinaster* , regardless of health status. The sensitivity of iWUE to CO_2_ in *P. pinea* temporally decreased since 1995 but later recovered and exceeded previous iWUE levels. The lack of response of iWUE in *P. pinaster* to CO_2_ after the extreme 1995 drought, particularly in non‐healthy trees, despite increasing drought stress, suggests a decrease in its plasticity to CO_2_ (Corcuera et al. 2010) which likely contributed to the species growth decline and the currently observed accelerated mortality in our study area. These results in adult trees agree with the little effect of CO_2_ on the water use efficiency of *P. pinaster* seedlings (Picon et al. 1996; Sánchez‐Gómez et al. 2017) and with *P. pinaster* 's low plasticity in populations under high stress (Ramírez‐Valiente et al. 2025). Several studies report recent decreasing responses of iWUE to CO_2_ in temperate forests (Adams et al. 2020; Belmecheri et al. 2021; Peñuelas et al. 2008; Waterhouse et al. 2004). However, a similar response in drought‐prone ecosystems, such as our studied Mediterranean forest, suggests that future climate changes may limit *P. pinaster* 's ability to adapt to increasing drought conditions.

Constant iWUE results from changes of similar magnitude in photosynthetic rates (A) and stomatal conductance (g_s_). Considering the high drought stress producing the long‐term growth decline in *P. pinaster* and its high mortality rates, increases in both A or g_s_ are unlikely (Aranda et al. 2024; Kannenberg et al. 2021). Constant iWUE suggests that non‐healthy *P. pinaster* trees may have reached their maximum capacity to control transpiration through stomatal conductance (g_s_) particularly in heavily defoliated and mistletoe‐infected trees (Meinzer et al. 2004; Sala et al. 2001; Zweifel et al. 2012). Shedding leaves, generally occurring after stomatal closure during droughts (Nadal‐Sala et al. 2021), would further reduce transpiration and improve carbon balance, as commonly older, less photosynthetically efficient leaves are dropped (Escudero and Mediavilla 2003). Drought‐induced defoliation would also imply reducing or maintaining g_s_ and similar changes in A (Guerrieri et al. 2019) particularly in the more isohydric species *P. pinaster* compared to *P. pinea* (Férriz et al. 2023). To compensate for extra water stress induced by mistletoe, non‐healthy *P. pinaster* most likely further decreased their g_s_ (Ehleringer et al. 1986; Meinzer et al. 2004; Zweifel et al. 2012), photosynthesis (Dobbertin and Rigling 2006), and crown‐level WUE to the point that ultimately trees were not able to maintain a positive carbon balance (Rigling et al. 2010; Zweifel et al. 2012). Leaf shedding may serve as a short‐term strategy to withstand drought stress, but extensive defoliation limits post‐drought recovery (Eilmann et al. 2013; Galiano et al. 2011) and may have initiated an irreversible decline spiral in *P. pinaster* which may have been exacerbated by increasing drought stress (McDowell et al. 2022; Sterck et al. 2024).

Both healthy and dead *P. pinea* showed greater capacity to regulate transpiration with increasing CO_2_ and drought than *P. pinaster* , which may allow the species to maintain its crown leaf area. Despite its low genetic variability, *P. pinea* exhibits high phenotypic plasticity of functional traits in response to drought stress, including iWUE (Sánchez‐Gómez et al. 2011). Although iWUE showed little increase in all *P. pinea* between 360 and 380 ppm CO_2_, it increased faster in dead trees than in healthy ones afterwards. Higher iWUE and lower growth in dead than healthy *P. pinea* could suggest long‐term stomatal constraints on carbon uptake and tree mortality (Saurer and Voelker 2022; Timofeeva et al. 2017) because of higher stomatal control limiting gas exchange and photosynthesis (Choat et al. 2012; Martin‐StPaul et al. 2017). Reduced xylem growth for ~10 years and the low anatomical plasticity to drought suggest that carbon constraints may have progressively reduced its hydraulic performance (Cailleret et al. 2017; Pellizzari et al. 2016; Puchi et al. 2021).

### 4.2 Differences in Species‐ and Health‐Specific Xylem Plasticity Under Climate Change Affect Mixed Forest Dynamics

Healthy individuals of both pine species had similar tracheid dimensions within the reported ranges for Mediterranean pine species (Carvalho et al. 2015; De Micco et al. 2007; Martin‐Benito et al. 2013, 2017). In contrast, *J. oxycedrus* tracheids were much smaller (particularly in the earlywood) than those in pines, consistent with results for other juniper species (Linton et al. 1998; Olano et al. 2012; Willson et al. 2008). These comparisons account for the conduit tapering with tree height, which influences axial trends in anatomical parameters (Anfodillo et al. 2006; Losso et al. 2018), particularly given the shorter stature of junipers compared to pines. Higher annual radial growth rates and potential hydraulic conductivity in pines reflect their higher productivity compared to junipers, provided enough soil water availability during the growing season. In contrast, narrower lumens in junipers, along with specific pit characteristics, reduce their vulnerability to xylem cavitation compared to coexisting pine species, increasing their resistance to hydraulic failure (Choat et al. 2008; Pittermann et al. 2006; Willson et al. 2008). This likely enhances juniper competitiveness in drought‐limited environments and under increasing climate stress (Breshears et al. 2009; Camarero et al. 2010; Gaylord et al. 2013).

Anatomical traits in the two pine species showed different temporal patterns, similar to the longer growth decline prior to mortality in *P. pinaster* (> 20 years) compared to *P. pinea* (~10 years) (Férriz et al. 2021), suggesting species‐specific differences in functional adjustment during their mortality processes. Healthy *P. pinaster* maintained higher earlywood LA than non‐healthy trees for the 40‐year period studied, but particularly after the 1995 drought. Wider LA suggests that healthy *P. pinaster* trees maintained higher turgor than unhealthy trees (Cabon et al. 2020; Peters et al. 2021; Steppe et al. 2006) during the growing season (Zweifel et al. 2006) when canopy transpiration is higher (Sánchez‐Costa et al. 2015). Wider lumens confer healthy *P. pinaster* trees higher potential xylem hydraulic conductivity in agreement with results in other conifers (Hereş et al. 2014; Pellizzari et al. 2016). Lower hydraulic conductivity and declining radial growth of non‐healthy trees years or decades before death suggest a deterioration of their xylem hydraulic performance and reduced capacity to support canopy transpiration (Dickman et al. 2015; Guerin et al. 2020; McDowell et al. 2008; Pellizzari et al. 2016). Higher growth rates and wider tracheid lumens in healthy trees over long periods of time may also increase sapwood water capacitance, which may provide water to maintain hydraulic performance under drought and contribute to tree survival (Barnard et al. 2011; Hölttä et al. 2009; Preisler et al. 2022). Similar variability in xylem anatomical traits and radial growth patterns before death in dead and declining *P. pinaster* suggests that declining trees had surpassed a physiological threshold and experienced negative legacies from which they were not able to recover (Férriz et al. 2021; Gea‐Izquierdo et al. 2014, 2021; Hammond et al. 2019), particularly under recent increases in drought stress in the area.

Wood anatomical traits expressed diverging temporal patterns and different responses to climate among different species and health statuses. In general, xylem functional traits in *P. pinaster* showed a stronger response to climate than those in *P. pinea* in agreement with the higher capacity of *P. pinea* to maintain leaf turgor and thus keep stable cell dimensions (Aranda et al. 2024). Healthy *P. pinaster* were more sensitive than non‐healthy trees, contrary to previous results for less drought‐tolerant conifers (Pellizzari et al. 2016; Voltas et al. 2013). During extreme droughts, lumen areas in *P. pinaster* were similar among different health statuses, but during wet years, LA increased only in healthy *P. pinaster,* thus increasing their hydraulic conductance. A decrease in tracheid LA during drought is a common response in *P. pinaster* (Carvalho et al. 2015; Vieira et al. 2014) and other conifers (Bryukhanova and Fonti 2013; Martin‐Benito et al. 2013; Sterck et al. 2008), driven by decreases in turgor pressure with the increase in water stress (Cabon et al. 2020; Steppe et al. 2006; Zweifel et al. 2006). Additionally, low summer moisture availability increased tracheid CWT of *P. pinaster* in the earlywood, but decreased it in the latewood, particularly in healthy trees. Wall thickness in *P. pinea* showed similar sensitivity to drought, particularly in the earlywood. The phenotypic plasticity in *P. pinaster* xylem reducing embolism under drought (Corcuera et al. 2011; Lamy et al. 2013) may contribute to the survival of some individuals but not others, particularly in more xeric‐adapted populations (Ramírez‐Valiente et al. 2025). These changes in response to climate occur mainly in the earlywood, which contributes most to the water conductance in the tree, highlighting the ability of healthy trees to adapt their xylem to maintain their hydraulic function (Férriz et al. 2023).

High defoliation in non‐healthy *P. pinaster* most likely reduced total C assimilation, as suggested by their lower growth and NSC levels compared to healthy trees (Galiano et al. 2011; Garcia‐Forner et al. 2016). Lower photosynthetic rates or reduced carbohydrate allocation to cell walls, prioritizing hydraulic conductance over xylem reinforcement to maintain canopy transpiration (Bryukhanova and Fonti 2013; Eilmann et al. 2009) may also explain the reduced CWT in dead trees (Cartenì et al. 2018; De Micco et al. 2019; P. Fonti et al. 2013). Wood density, related to the CWT:LA ratio, and xylem tracheid density, i.e., tracheid number per area (Song et al. 2022) are often associated with increased resistance to embolism at the inter‐ and intraspecific levels in gymnosperms, including *P. pinaster* (Anderegg et al. 2016; Bouche et al. 2014; Hacke et al. 2001; Nabais et al. 2018). Long‐term impairment in carbon fixation or mobilization would reduce their ability to adapt to varying conditions and reduce their post‐drought resilience, as shown by our results. The higher xylem plasticity observed in healthy trees likely allowed them to capitalize on periods of higher water availability to sustain growth and productivity, while adjusting their wood anatomy to withstand periods of low water availability, as those observed in our study area since the 1980s.

Regardless of the exact mechanism contributing to *P. pinaster* decline, the different temporal scales, syndromes, and environmental factors involved compared to *P. pinea* will have important implications for forest dynamics under climate change. As *P. pinea* benefits from rising CO_2_ by increasing its iWUE and adjusting it to drought variability more than *P. pinaster* , the decline in this latter species may continue, particularly under warmer and drier future conditions, while *P. pinea* is able to persist. Different mortality rates between species may result in important composition shifts (Martínez‐Vilalta and Lloret 2016), especially considering that *P. pinaster* , the most abundant species in the area, also experiences a lack of regeneration (Gea‐Izquierdo et al. 2019).

## 5 Conclusions

As one of the many drought‐prone biomes globally, Mediterranean forest ecosystems face unique but representative challenges under a changing climate. Our study highlights the functional species‐specific responses of coniferous trees to prolonged drought stress and increasing CO_2_, emphasizing the critical role of functional trait plasticity in determining tree survival and mortality under global change. Long‐term decline in the less drought‐tolerant *P. pinaster* was related to the combination of low plasticity in anatomical traits and a decreased sensitivity to rising CO_2_ due to legacy and direct effects of drought stress, compounded by biotic factors such as mistletoe infestation. With limited plasticity in xylem and stomatal adjustment, unhealthy *P. pinaster* trees may have been unable to adapt to changing growing conditions, contributing to their decline and mortality. The similarities between healthy and non‐healthy trees of this species may anticipate further decline at the regional level. The limited anatomical plasticity shown by *P. pinea* regardless of health status, but the higher response to CO_2_ in dead trees, suggests important differences in the mortality process between both pine species. Interspecific differences in the coordination of the functional responses of xylem hydraulic anatomical traits and stomatal control under drought stress may explain the temporally contrasting mortality process in the phylogenetically and ecologically close species *P. pinaster* and *P. pinea* and the increasing success of the more plastic junipers. These differences in physiological and anatomical responses underscore the diverse strategies trees use to cope with environmental stress, suggesting that species or individuals with higher plasticity in stomatal and xylem traits may be more resilient to drought.

## Author Contributions

**Dario Martin‐Benito:** conceptualization, data curation, formal analysis, funding acquisition, investigation, methodology, project administration, resources, software, validation, visualization, writing – original draft. **Macarena Férriz:** conceptualization, data curation, formal analysis, investigation, methodology, writing – original draft. **María Conde:** methodology, writing – review and editing. **Georg von Arx:** methodology, resources, writing – review and editing. **Patrick Fonti:** methodology, resources, writing – review and editing. **José Miguel Olano:** methodology, resources, writing – review and editing. **Guillermo Gea‐Izquierdo:** conceptualization, funding acquisition, methodology, project administration, resources, writing – review and editing.

## Conflicts of Interest

The authors declare no conflicts of interest.

## Supporting information


Data S1.

## Data Availability

The data that support the findings of this study are openly available in Dryad at https://doi.org/10.5061/dryad.8cz8w9h3p.
