# Supplementary material for: Loss of Stomatal Regulation Sensitivity to CO2 and Reduced Xylem Hydraulic Conductivity Contribute to Long‐Term Tree Decline and Mortality
Source: Glob Chang Biol. 2025 May 20;31(5):e70221. doi: 10.1111/gcb.70221 (PMC12090040; doi:10.1111/gcb.70221)
Supplement: Supplementary file 1 — Data S1. [file GCB-31-e70221-s001.pdf]

Supporting information for:

Loss of stomatal regulation sensitivity to CO_2 and reduced xylem hydraulic conductivity contribute to long-term tree decline and mortality

Dario Martin-Benito[1,*], Macarena Férriz[1, 2], María Conde[1], Georg von Arx[3,4], Patrick Fonti[3], José Miguel Olano[5], Guillermo Gea-Izquierdo[1]

[1] Institute of Forest Sciences ICIFOR, INIA-CSIC, Ctra. La Coruña km 7.5, 28040 Madrid, Spain

[2] Department of Geography, Indiana University, Bloomington, IN, USA.

[3] Swiss Federal Institute for Forest, Snow and Landscape Research WSL, Zürcherstrasse 111, 8903 Birmensdorf, Switzerland

[4] Oeschger Centre for Climate Change Research, University of Bern, Falkenplatz 16, 3012, Bern, Switzerland

[5] iuFOR, EiFAB, Campus de Soria, Universidad de Valladolid, E-42004 Soria, Spain

*, corresponding author: Dario Martin-Benito (dmartin@inia.csic.es)

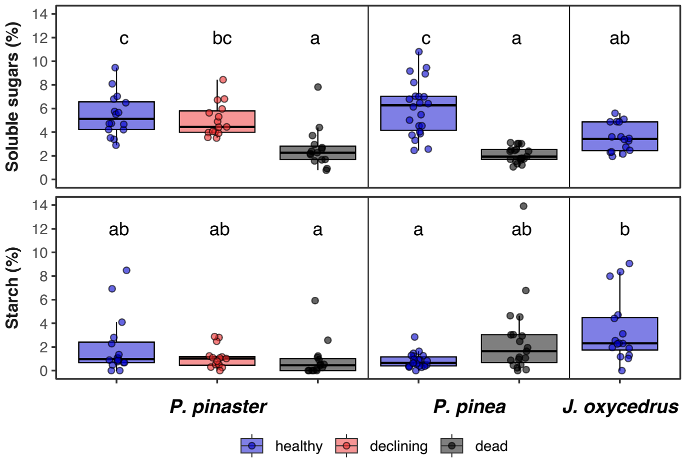

Figure S1. Relative content in non-structural carbohydrates (NSC) separated into soluble sugars (top) and starch (bottom). For each compound group, different letters show significant differences between species and health status (p≤0.05). Samples size is 5 trees per species and health status in all cases.

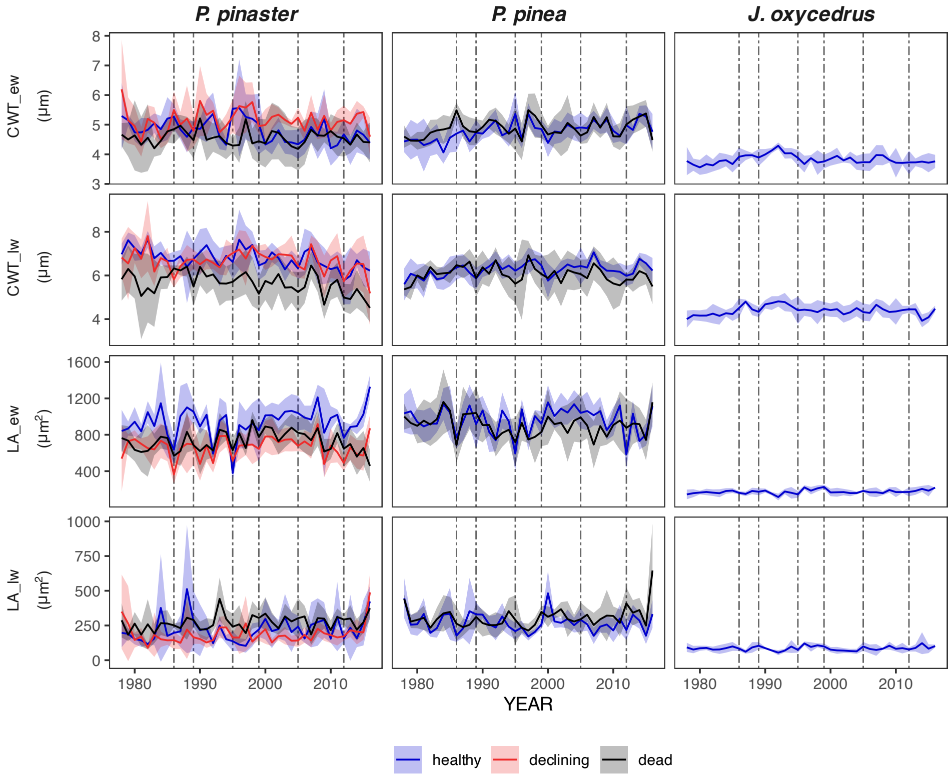

Figure S2. Annual anatomical variables for the period 1978-2016. Lines represent average values and shaded areas represent confidence intervals around the mean (± 1.96·SEM, standard error of the mean). Vertical dashed lines show the 6 driest years (defined as lowest August SPEI12, see Figure 1): 1986, 1989, 1995, 1999, 2005, 2012. CWT, cell wall thickness; LA, lumen area. EW and LW stand for early- and late-wood, respectively. Samples size was 5 trees per species and health status in all cases.

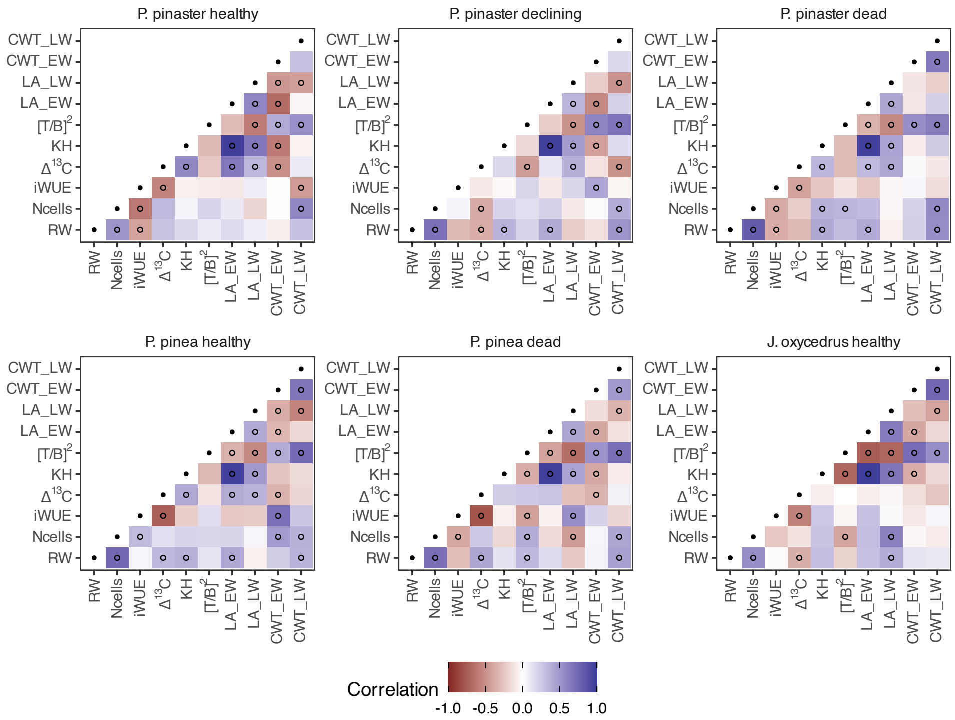

Figure S3. Cross-correlation coefficients among growth (ring-width), xylem anatomical traits and isotopic variables (iWUE and Δ^{13}C) for the period 1978-2016. CWT, cell-wall thickness; LA, lumen area; $[T/B]^2$, resistance-to-implosion factor; KH; theoretical xylem hydraulic conductivity; Δ^{13}C, discrimination against ^{13}C; iWUE, intrinsic water use efficiency; Ncells, radial number of cells; RW, ring width. EW and LW stand for early and latewood, respectively. Significant correlations ($p \leq 0.05$) are highlighted with empty circles. Black dots show the matrix diagonal where r=1.

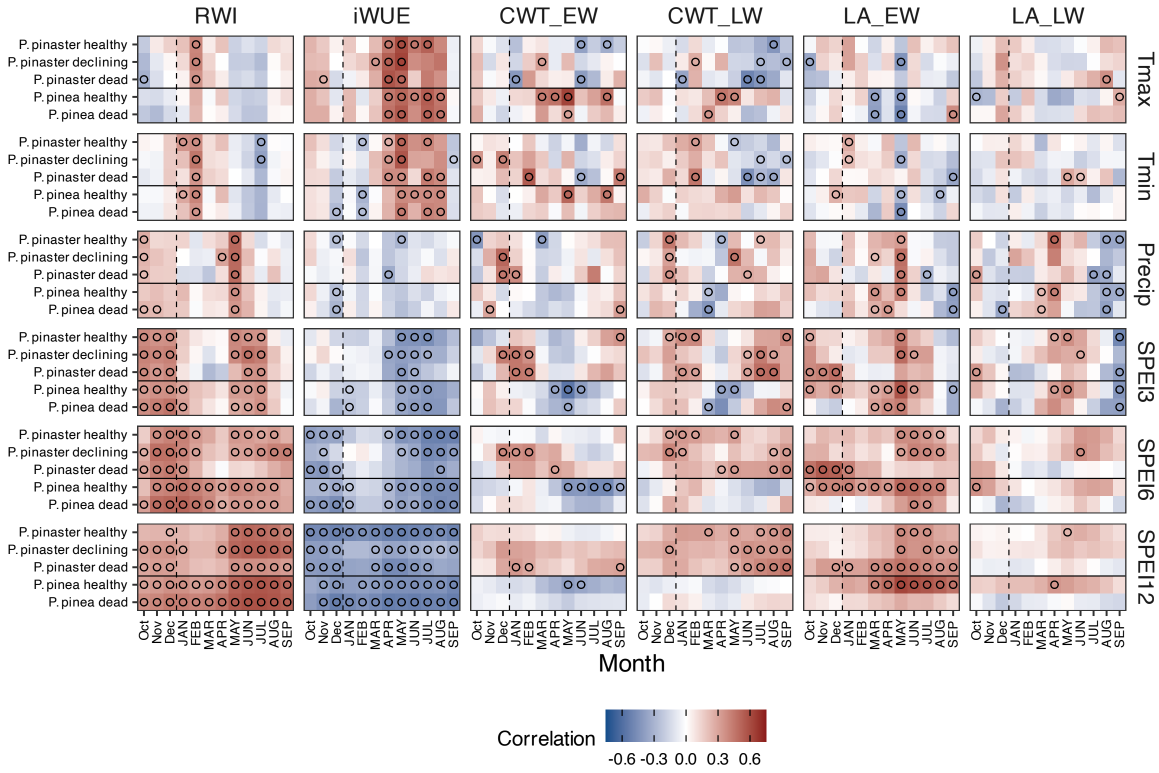

Figure S4. Climate influence on growth and anatomical variables. Correlations between ring and anatomical variables with monthly climate variables from October of the previous year to September of the current year for the period 1978-2016. Significant correlations ($p \leq 0.05$) are highlighted with dots. RWI, ring width index; iWUE, intrinsic water-use efficiency; CWT cell wall thickness; LA lumen area. EW and LW stand for early and latewood, respectively. Significant correlations ($p \leq 0.05$) are highlighted with circles.

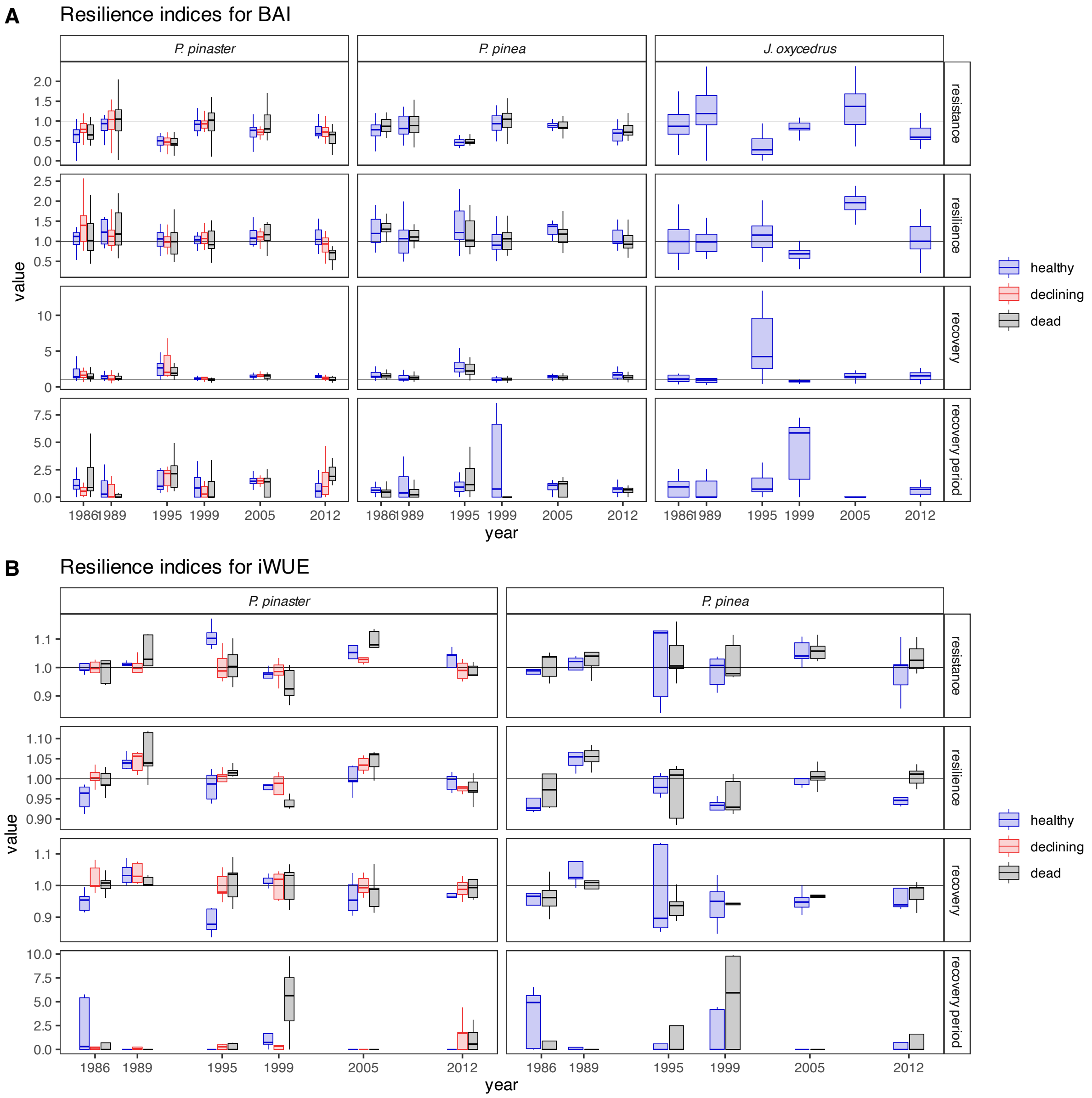

Figure S5. Analyses of resilience indices for basal area increment (BAI) and intrinsic water use efficiency (iWUE) for the 6 drought years analyzed. BAI and iWUE are normalized for comparison. Drought years identified as those with lowest August SPEI12 between 1978 and 2016 (see Figure 1). Statistics for the boxplots were calculated using all data, but outliers were excluded in this figure for clarity.

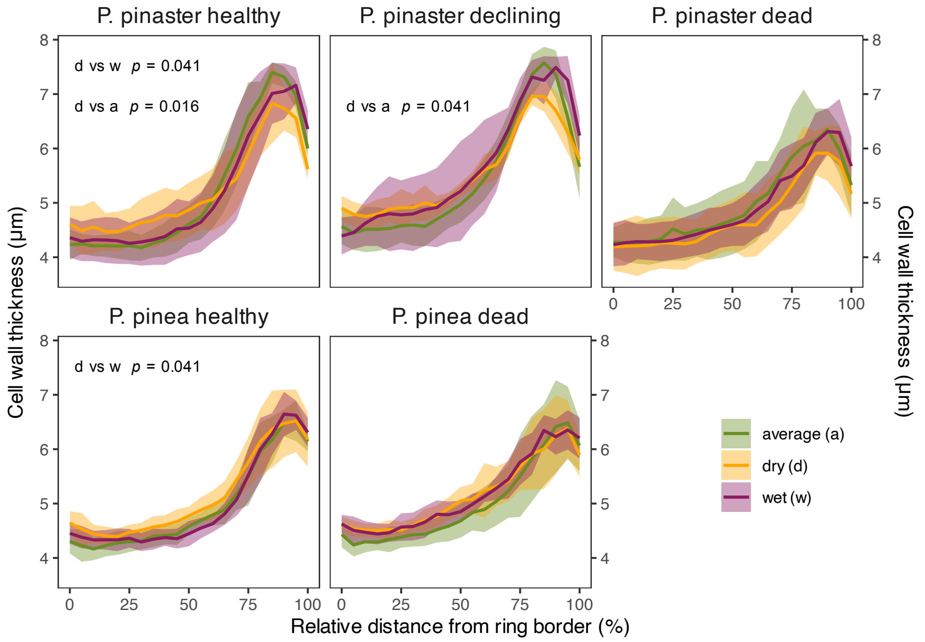

Figure S6. Variation of tracheid cell-wall thickness (CWT) along the annual growth ring of both pine species for the 6 driest, 6 wettest and 6 average years i.e. those closest to zero (estimated from August SPEI 6 months between 1978 and 2016; see Figure 1). Thick lines represent average values and shaded areas represent confidence intervals around the mean (± 1.96·SEM, standard error of the mean). Kolmogorov-Smirnoff tests were used to check for significant differences between results for dry (d), average (a) and wet (w) years within each species and health status combination. Results shown when significant for two-sample Kolmogorov-Smirnov tests ($\alpha \leq 0.05$).

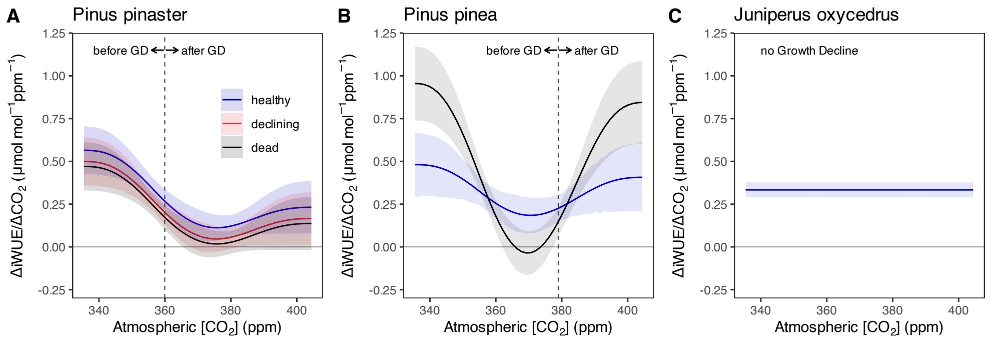

Figure S7. Slopes of iWUE response to atmospheric CO_2 concentration (ΔiWUE/ΔCO_2) for each species and health status. Slopes are estimated as the first derivative of each smooth function for the considered parameter (atmospheric CO_2). Shaded areas represent the 95% simultaneous confidence intervals. Levels of atmospheric CO_2 for which the confidence intervals do not include 0, show significant temporal changes in the iWUE response.

Table S1. Xylem anatomical variables measured for each species and health status. Values are means of 5 trees per species and health combination averaged over the full period (1978-2016). Standard deviations in parenthesis. Variables are: CWT, cell-wall thickness; LA, lumen area; DH, mean hydraulic diameter; KH; theoretical xylem hydraulic conductivity; $(T/B)^2$, resistance-to-implosion factor; Ncells, radial number of cells; $\Delta^{13}C$, discrimination against ^{13}C; iWUE, intrinsic water use efficiency. Section, either earlywood (EW), latewood (LW) or the entire ring. Different letters indicate significant differences between means within the same variable and ring section using linear mixed effects models to account for the lack of independence of data within each individual and temporal autocorrelation. Tree height was also included in these mixed models to account for the potential influence of tree height on xylem anatomical variables.

		Pinus pinaster			*Pinus pinea*		*J. oxycedrus*
**Variable**	**Section**	**Healthy**	**Declining**	**Dead**	**Healthy**	**Dead**	**Healthy**
**CWT (µm)**	EW	4.81 b (0.71)	5.17 b (0.69)	4.57 b (0.69)	4.8 b (0.53)	4.89 b (0.55)	3.83 a (0.3)
	LW	6.77 c (0.87)	6.67 c (0.88)	5.68 b (1.03)	6.26 bc (0.57)	6.05 bc (0.8)	4.39 a (0.4)
**LA (μm^2)**	EW	936.77 d (260.95)	664.51 b (194.86)	730.72 bc (192.48)	961.78 d (224.43)	905.7 cd (248.19)	172.42 a (42.58)
	LW	221.76 bc (174.97)	184.07 b (109.79)	270.28 cd (107.08)	271.27 cd (111.26)	308.42 d (128.33)	84.68 a (30.82)
**Kh (10^{-12}, kg m^{-1} s^{-1} MPa^{-1})**	EW	41.86 d (20.23)	21.46 b (10.53)	25.02 bc (11.30)	42.99 d (17.96)	39.87 cd (20.00)	13.15 a (0.58)
	LW	4.18 bc (7.92)	2.30 b (3.39)	4.12 bc (3.37)	4.12 bc (3.92)	5.34 c (6.18)	0.35 a (0.27)
**$(T/B)^2$**	EW	0.16 ab (0.19)	0.23 b (0.27)	0.13 a (0.13)	0.09 a (0.06)	0.11 a (0.08)	0.24 b (0.09)
	LW	1.08 c (0.85)	0.80 bc (0.61)	0.47 ab (0.37)	0.40 a (0.26)	0.35 a (0.18)	0.60 ab (0.30)
**Ncells**	EW	29.88 a (13.77)	22.4 a (20.19)	36.04 a (26.21)	22.08 a (14.72)	31.58 a (22.92)	60.27 b (36.9)
	LW	9.77 ab (5.19)	7.03 a (6.14)	11.67 ab (7.70)	7.69 a (4.76)	11.03 ab (8.27)	16.24 b (10.5)
**iWUE ($\mu mol\ mol^{-1}$)**	Ring	100.01 ab (8.31)	102.17 ab (7.31)	100.48 ab (8.55)	95.38 a (9.59)	96.31 ab (9.88)	108.39 b (7.88)

Table S2. Summary of generalized additive models (GAM) tested for evaluating the effects of health status, atmospheric CO_2 concentration, and drought (August SPEI12) on intrinsic water-use efficiency (iWUE) in *P. pinaster* and *P. pinea*. Models were compared based on their ΔAIC, deviance explained, adjusted R^2, and the number of parameters used. Models in bold were selected. See Table S2 for more details on selected models. s() function represents smoothing (non-linear) terms, the by= Status and by=period arguments mean that the smooth term for CO_2 or SPEI12 can vary between different levels status or period. Dev. Exp, deviance explained: Adj. R^2, adjusted R^2: No. Params, number of parameters.

Species	Model	Equation	ΔAIC	Dev. Exp (%)	Adj. R^2	No. Params
P. pinaster	1	iWUE ~ Status + s(CO_2) + s(SPEI12)	9.24	0.318	0.311	9
	2	iWUE ~ Status + s(CO_2) + s(SPEI12) + s(CO_2, by = Status)	3.68	0.329	0.320	18
	**3**	**iWUE ~ Status + s(CO_2) + s(CO_2, by = Status,) + s(SPEI12, by = Status)**	**0.00**	0.334	**0.322**	**24**
	4	iWUE ~ Status + s(CO_2) + s(SPEI12) + s(CO2, by = Status) + s(SPEI12, by = Status) + s(SPEI12, by = period)	3.59	0.338	0.325	36
	5	iWUE ~ Status + s(CO_2) + s(SPEI12, by=period) + s(CO_2, by=Status) + s(SPEI12, by= (Status, period))	2.05	0.344	0.330	45

Species	Model	Equation	ΔAIC	Dev. Exp (%)	Adj. R^2	No. Params
P. pinea	1	iWUE ~ Status + s(CO_2) + s(SPEI12)	3.77	0.589	0.583	8

**2**	**iWUE ~ Status + s(CO_2) + s(SPEI12) + s(CO_2, by = Status)**	0.00	0.595	0.588	**14**
3	iWUE ~ Status + s(CO_2) + s(CO_2, by = Status) + s(SPEI12, by = Status CO_2)	1.99	0.595	0.587	17
4	iWUE ~ Status + s(CO_2) + s(SPEI12) + s(CO_2, by = Status) + s(SPEI12, by = Status) + s(SPEI12, by = period)	3.62	0.601	0.590	21
5	iWUE ~ Status + s(CO) + s(SPEI12, by=period) + s(CO_2, by=Status) + s(SPEI12, by= (Status, period))	3.62	0.601	0.590	23

Table S3. Summary for the generalized additive models (GAM) fit to the estimate the effect of atmospheric CO_2 concentration on iWUE simultaneously accounting for the effect of drought (August SPEI12). In smooth terms, *edf* stands for the estimated degrees of freedom of each parameter.

Species	Term				
P. pinaster	**Parametric coefficients**	**Estimate**	**SE**	**t-value**	**p-value**
	Intercept (Dead)	100.51	0.48	209.20	<0.001
	Status (Declining)	1.64	0.68	2.42	0.016
	Status (Healthy)	-0.50	0.68	-0.73	0.463
	**Smooth terms**	**edf**		**F-statistic**	**p-value**
	CO_2	2.79		22.43	<0.001
	CO_2 x Status (Dead)	$3.94 \cdot 10^{-4}$		0.072	0.994
	CO_2 x Status (Declining)	1.00		0.670	0.413
	CO_2 x Status (Healthy)	1.00		7.169	0.008
	SPEI12 x Status (Dead)	1.00		1.999	0.111
	SPEI12 x Status (Declining)	1.00		0.399	0.527
	SPEI12 x Status (Healthy)	1.00		11.86	<0.001
	**Num. Observations**				585
	**R^2 (adjusted)**				0.32
	**Deviance explained (%)**				33.6%
P. pinea	**Parametric coefficients**	**Estimate**	**SE**	**t-value**	**p-value**
	Intercept (Dead)	96.33	0.43	221.71	<0.001
	Status (Healthy)	-1.49	0.61	-2.43	0.0156
	**Smooth terms**	**edf**		**F-statistic**	**p-value**
	CO_2	2.56		27.88	<0.001
	SPEI12	1.00		37.79	<0.001
	CO_2 x Status (Dead)	1.82		6.76	0.0021
	CO_2 x Status (Healthy)	1.00		14.17	<0.001

	**Num. Observations**				380
	**R² (adjusted)**				0.59
	**Deviance explained (%)**				60.00%
J. oxycedrus	**Parametric coefficients**	**Estimate**	**SE**	**t-value**	**p-value**
	Intercept (Healthy)	108.02	0.501	213.1	<0.001
	**Smooth terms**	**edf**		**F-statistic**	**p-value**
	CO_2	1.00		161.89	<0.001
	SPEI12	1.57		0.158	0.681
	**Num. Observations**				47
	**R² (adjusted)**				0.79
	**Deviance explained (%)**				79.80%
